# Supporting Recovery College trainers: a qualitative study on complementary knowledge in Quebec and Lombardy

**DOI:** 10.3389/fpsyt.2025.1702341

**Published:** 2025-12-10

**Authors:** Martine Vallarino, Filippo Rapisarda, Laurence Cournoyer, Anick Sauvageau, Brigitte Vachon, Gianpaolo Scarsato, Paolo Cacciani, Antonio Vita, Catherine Briand

**Affiliations:** 1 Department of Occupational Therapy, University of Quebec at Trois-Rivières, Trois-Rivières, QC, Canada; 2 Research Center of Institut universitaire en santé mentale de Montréal, Montreal, QC, Canada; 3 School of Readaptation, University of Montreal, Montreal, QC, Canada; 4 Azienda Socio Sanitaria Territoriale degli Spedali Civili di Brescia, Dipartimento di Salute Mentale e delle Dipendenze, Brescia, Italy; 5 Universita Degli Studi di Brescia, Dipartimento di Scienze Cliniche e Sperimentali, Brescia, Italy

**Keywords:** Recovery College, training program, trainers, co-production, mental health

## Abstract

**Background:**

Recovery Colleges (RCs) are educational centers offering free courses on mental health, well-being, recovery, and living well together. They represent an innovative approach to mental health, going beyond clinical and therapeutic interventions to foster constructive dialogue between people with lived experience and professionals with theoretical or clinical knowledge. Fidelity to the RC model, particularly to the principle of co-production, is considered essential to ensure quality. However, despite the crucial role of trainers for maintaining alignment with RC principles and values, little research has examined how trainers could be trained and supported to coproduce RC courses. This study aimed to explore the experiences of RC trainers and coordinators, describing challenges and good practices encountered in working with complementary types of knowledge.

**Methods:**

A qualitative exploratory multicenter design was adopted. Data were collected between May and December 2024 through five online focus groups involving trainers and coordinators from two RCs, one in Quebec, Canada, and one in Lombardy, Italy. Verbatim transcripts were analyzed using a stepwise thematic analysis.

**Results:**

Twenty-seven people with diverse backgrounds participated in the study. Eight main themes (and their respective subthemes) emerged from participants’ narratives: the distinctive nature of the RC model which requires the embodiment of its values; the development of core competencies such as knowledge integration, mobilization of experiential knowledge, and group facilitation skills; the dynamic within the trainers’ dyads, described as a relational process based on mutual trust and negotiation; strengths and challenges of the co-production process within the dyad and with learners; ongoing activities and tools to ensure trainers’ alignment with the model and activities to support the trainer’s role.

**Discussion:**

Results suggest the importance of raising awareness among trainers about relevant elements to be considered in designing and implementing a RC training program. It is therefore important to foster egalitarian and supportive relationships in the trainers’ dyad, as these can serve as a model for co-production during RC courses. Finally, to improve knowledge complementarity, trainers need to receive continuous support, through ongoing training activities and other learning opportunities to ensure alignment with value and principles of RC model.

## 1 Introduction

Established in England in 2009 and now spread worldwide, Recovery Colleges (RCs) are educational hubs that provide free courses on mental health, well-being, recovery, and living well collectively (1). RCs propose a new paradigm based on principles of mutual learning, egalitarianism and recognition of experiential knowledge (2). The distinctive feature of the RC model is its emphasis on the complementarity of experiential, clinical, and theoretical knowledge (3). Within each training course, the various types of knowledge are embodied by trainers and learners from different backgrounds (people with mental illness, family members, professionals, healthcare workers, citizens).

The RC model represents, first and foremost, an innovation in the approach to mental health; it differs from a clinical-therapeutic approach in favor of a form of constructive dialogue between individuals with experiential knowledge and professionals with a theoretical or clinical background (1, 4, 5). Several literature contributions have identified fidelity to the theoretical RC model, in terms of foundational values and principles, as a crucial aspect of RC implementation to ensure its quality (2, 6). Among these, the concept of co-production emerged as a central element of the RC model in a worldwide survey involving RCs in 22 countries (3). Co-production involves the integration of lived experience and professional expertise at every stage—from course design to delivery—through continuous collaboration that shapes curriculum, operations, and quality. As an embodiment of co-production, every RC course is co-developed and co-facilitated by a dyad of trainers—one with lived experience and another with a professional or theoretical background—who work with complementary types of knowledge. RC context fosters mutual learning, shared responsibility, hope and transforming education into an inclusive, value-driven and sustainable process focused on empowerment and meaningful change (1, 5, 7–10).

A recent scoping review was conducted to examine co-production and identify practical examples of training, guidance, and/or support provided to trainers, enabling them to co-design and co-deliver RC courses (11). Authors identified 19 research contributions, mainly conducted in the UK and Australia between 2013–2024, which reported pilot studies and case studies on trainers’ experiences in supporting co-production. The results converge in highlighting the absence of specific training programs on the topic of co-production, although some activities to support co-production are proposed, such as group meetings and peer debriefings (12–18). Although there is unanimous consensus on the importance of trainers following a specific training program that emphasizes the co-production dimension, several authors have argued that there are few documented experiences (3, 19, 20). In fact, there is still little research on how to develop training programs for RC trainers that support the development of competencies related to the complementarity of expertise, the integration of different types of knowledge, and co-production (11). A recent study depicted the logical model of the Train-the-Trainer (TTT), providing an example of how a TTT program can be designed and successfully implemented (21). Additionally, at the conclusion of a systematic review, Briand and colleagues (22) emphasized the need for future studies to document training programs, tools, and strategies for developing and sustaining RC trainers’ competencies, as well as to ensure the quality and fidelity of the model. According to several other authors, the experience and involvement of trainers in RC studies are not yet sufficiently explored, even though these individuals play a crucial role not only in the effective implementation of RC, but as agents of broader organizational and social change (8, 23, 24).

The aim of this study is therefore to fill this gap by exploring issues of knowledge complementarity, while describing the challenges and good practices encountered by trainers and coordinators. To achieve this aim, the research has the following objectives: 1) identify the most relevant elements to be considered in the training of trainers; 2) explore trainers’ experience of working with complementary expertise; 3) explore strengths and challenges of working with complementary expertise; 4) identify strategies and tools to support complementarity of expertise during the initial and ongoing training of trainers.

## 2 Method

### 2.1 Study design and research context

This qualitative explorative study was conducted in multiple locations adopting a multicenter data collection in Quebec (Canada) and Lombardy (Italy). In Quebec, participants were recruited among trainers and coordinators of the Health and Recovery Learning Center (Centre d'Apprentissage Santé et Rétablissement – CASR), the only French-language RC in Canada established in 2019. At the time of data collection, CASR provided over 200 free online courses, each lasting six hours (three two-hour sessions), which reached more than 4,000 learners from diverse backgrounds. CASR governance is multi-partner and multi-sectoral (including health, education, community, civic, and research). A variety of topics were covered, including recovery, stigma, well-being and mental health, social networks and support, workplace mental health, social inclusion and living better together. In Lombardy, participants were recruited from trainers and coordinators of the CoLab Torre Cimabue project of the Department of Mental Health and Substance Abuse of Spedali Civili Hospital Trust in Brescia. The project was established in 2014 and was the first RC developed in Italy offering free courses (also known as Corsi FOR-Formazione e Opportunità per la Recovery) to learners with different types of knowledge and diverse backgrounds (family members, individuals with mental illness, mental health and social workers, university students, citizens). To date, the CoLab has provided 323 free courses, each lasting six hours (three two-hour sessions), which have reached over 4,000 learners since the beginning of its activity. The decision to collect data in these two contexts was driven by several factors. First of all, in addition to offering free RC courses to the general population, both RCs have two well-established training programs for becoming RC trainers. Collecting qualitative data from two different geographical areas can improve the validity and generalizability of the results, while also reducing the risk—particularly common in qualitative research—of producing findings that are only applicable to a specific local context. These two RCs share several key similarities: both have solid experience in the field, the staff have received training and supervision from the founders of the original English RC, and they have maintained fidelity to the model. In addition, both programs have implemented a catalog of over twenty courses, reaching a diverse group of learners. Finally, both RCs are aligned with the fidelity matrix criteria of the Nottingham Recovery College (10); therefore, courses are co-designed and co-facilitated by a dyad of trainers with diverse, clinical, theoretical, and experiential knowledge.

### 2.2 Sampling and recruitment

In this qualitative study, participants were selected based on minimal eligibility criteria established in collaboration with staff from the two RCs located in Quebec (QC) and Lombardy (LO). Eligible participants had to be at least 18 years old, able to participate in an online meeting (access to technology and basic computer skills), have co-facilitated at least one RC course as a trainer, or be engaged in RC as a coordinator. The perspectives of both trainers and coordinators were sought. No exclusion criteria were applied. A convenience sampling approach was adopted to recruit individuals with relevant experience who were accessible to the research team. The principal investigator then asked the coordinators of the two RCs to invite all trainers and coordinators to participate in the research to provide an adequate and diverse representation of participants’ profiles in terms of gender, age, and type of knowledge. Socio-demographic data were collected via a brief pre-consent questionnaire. The questionnaire was designed to inform group composition and to capture self-identification, with participants selecting one or more categories from a-list of option and indicating their type(s) of knowledge (multiple responses permitted). Owing to the level of detail, the full distribution is presented in **Table 1** and can be consulted using individual participant codes. Once the sample was identified, trainers and coordinators were invited to participate in focus groups for data collection.

**Table 1:** Description of the participants’ sample.

Participant	Sample	Role	Gender	Age	Education	Self-identification as	Years of RC exp	Type of knowledge
P1 FG1	QC	T	Female	40-59	MD	Education sector professional	5	THEO
P2 FG1	QC	T	Male	20-39	BD	College or university student	2	EXP, THEO
P3 FG1	QC	C	Female	40-59	BD	Health and social service worker	1	EXP, CLIN, THEO
P4 FG1	QC	T	Female	60+	HS	ASME & Family member	1	EXP
P1 FG2	QC	T	Female	40-59	BD	Administrative staff, manager, executive	5	EXP, CLIN, THEO
P2 FG2	QC	T	Female	60+	BD	Peer worker, patient partner, person in recovery	10	EXP, THEO
P3 FG2	QC	C, T	Female	40-59	MD	Health and social service worker	2	EXP, CLIN, THEO
P4 FG2	QC	T	Male	60+	BD	Peer worker, patient partner, person in recovery	10	EXP, THEO
P5 FG2	QC	T	Female	40-59	BD	Other	6	EXP
P1 FG3	QC	C	Female	40-59	MD	Administrative staff, manager, executive	4	CLIN
P2 FG3	QC	T	Female	40-59	PhD	Other	1	EXP, CLIN, THEO
P3 FG3	QC	T	Male	60+	BD	Peer worker, patient partner, person in recovery	8	EXP, CLIN, THEO
P4 FG3	QC	T	Female	40-59	BD	HSSW & PPP	3	EXP, THEO
P1 FG4	LO	T	Female	20-39	BD	Peer worker, patient Partner, person in recovery	2	EXP
P2 FG4	LO	C, T	Female	40-59	MD	Health and social service worker	10	CLIN, THEO
P3 FG4	LO	T	Female	40-59	HS	PPP & Family member & Citizen	10	EXP
P4 FG4	LO	T	Male	40-59	MD	Health and social service worker	4	CLIN, THEO
P5 FG4	LO	T	Female	20-39	MD	Health and social service worker	1	CLIN, THEO
P6 FG4	LO	C, T	Male	40-59	BD	Health and social service worker	10	EXP, CLIN, THEO
P1 FG5	LO	C, T	Female	20-39	MD	Health and social service worker	9	CLIN
P2 FG5	LO	T	Female	20-39	Other	Peer worker, patient partner, person in Recovery	2	EXP
P3 FG5	LO	T	Female	60+	HS	Family member	4	EXP, THEO
P4 FG5	LO	T	Male	60+	Other	Family member & PPP	10	EXP
P5 FG5	LO	T	Female	40-59	BD	Community organization worker	5	CLIN
P6 FG5	LO	T	Female	20-39	HS	College or university student	1	THEO
P7 FG5	LO	T	Female	20-39	BD	Health and social service worker	5	CLIN, THEO
P8 FG5	LO	T	Female	60+	MD	Peer worker, patient partner, person in recovery	5	EXP

EXP, experience (in RC courses, as learner or facilitator); FG, focus group; QC, Quebec; LO, Lombardy; Role: T, Trainer; C, Coordinator; Education: MD, master’s degree; BD, Bachelor’s Degree; HS, High School; Self-identification; HSSW, health and social service worker; ASME, Administrative Staff, Manager, Executive; PPP, Peer worker, Patient Partner, Person in Recovery; Type of knowledge: EXP, experiential; CLIN, clinical; THEO, theoretical.

### 2.3 Data collection procedures

Data were collected through five focus groups conducted between May and December 2024, each lasting between 90 and 120 minutes. The sample included RC trainers and coordinators. Three focus groups were conducted in French with the CASR team by the principal investigator (MV) and a co-author of this article (AS), while two focus groups were conducted in Italian with the CoLab team by the principal investigator (MV) and a co-author of this article (FR). All focus groups were held online via the Zoom platform and were video recorded. The research project (number 2024-3642) was approved by the Research Ethics Committee of the Centre Intégré Universitaire de Santé et de Services Sociaux de l'Est-de-l’Île-de-Montréal (CIUSSS-EMTL). Each participant signed an informed consent form, before entering the study. To ensure privacy, video files and transcripts were stored on a secure server accessible only to members of the research team. Recordings were automatically transcribed, reviewed and corrected manually to address grammatical and content inaccuracies. All participant names were anonymized and replaced with unique study identifiers to ensure confidentiality. During the focus group, researchers used a semi-structured interview guide consisting of nine open-ended questions, designed to explore the four research objectives of the study. The interview structure was chosen by the research team in light of the recent literature described above. This approach allowed for both consistency across groups and the flexibility needed to explore emerging topics in depth.

### 2.4 Qualitative analysis

Verbatim transcripts were uploaded into NVivo 14 and analyzed using an iterative qualitative analysis approach. The analysis followed the stepwise method developed by Miles and Huberman (25), which consists of three key stages: (a) coding the data, (b) organizing codes into subthemes and subsequently into larger themes, and (c) validating the themes. A codebook was developed through an iterative process involving the principal investigator (MV) and two co-authors of this article (FR and CB), who collaboratively defined the themes and their definitions. Initial coding was conducted independently, and any discrepancies were resolved through discussion between the first two coders (MV and FR) until consensus was reached. Then, the third coder (CB) proceeded to a counter-analysis, to guarantee the exclusivity and clarity of themes and categories, the accuracy of quote representation, and the correct classification of quotes. Following this step, final improvements were made to strengthen the validity and transparency of the analytical process.

## 3 Results

### 3.1 Sample description

The sample consisted of 27 participants, 13 from Quebec (Canada) and 14 from Lombardy (Italy). Most of the participants (77.8%) were female, aged between 40 and 59 years (48.1%). Six participants had the role of coordinator, and, among them, four also facilitated courses as trainers. Participants had diverse educational backgrounds, most frequently bachelor’s degrees (44.4%) or master’s degrees (29.6%). Participants most often identified themselves as health and social service workers (33.3%) or as peer workers, patient partners, or people in recovery (33.3%). Experience as RC trainers ranged from 1 to 10 years, with an average of 5.0 years (standard deviation = 3.4). Participants described their knowledge as experiential (66.7%), theoretical (55.5%), and clinical (48.1%). Participants profiles are described in **Table 1**.

### 3.2 Emerged themes

**Figure 1:**
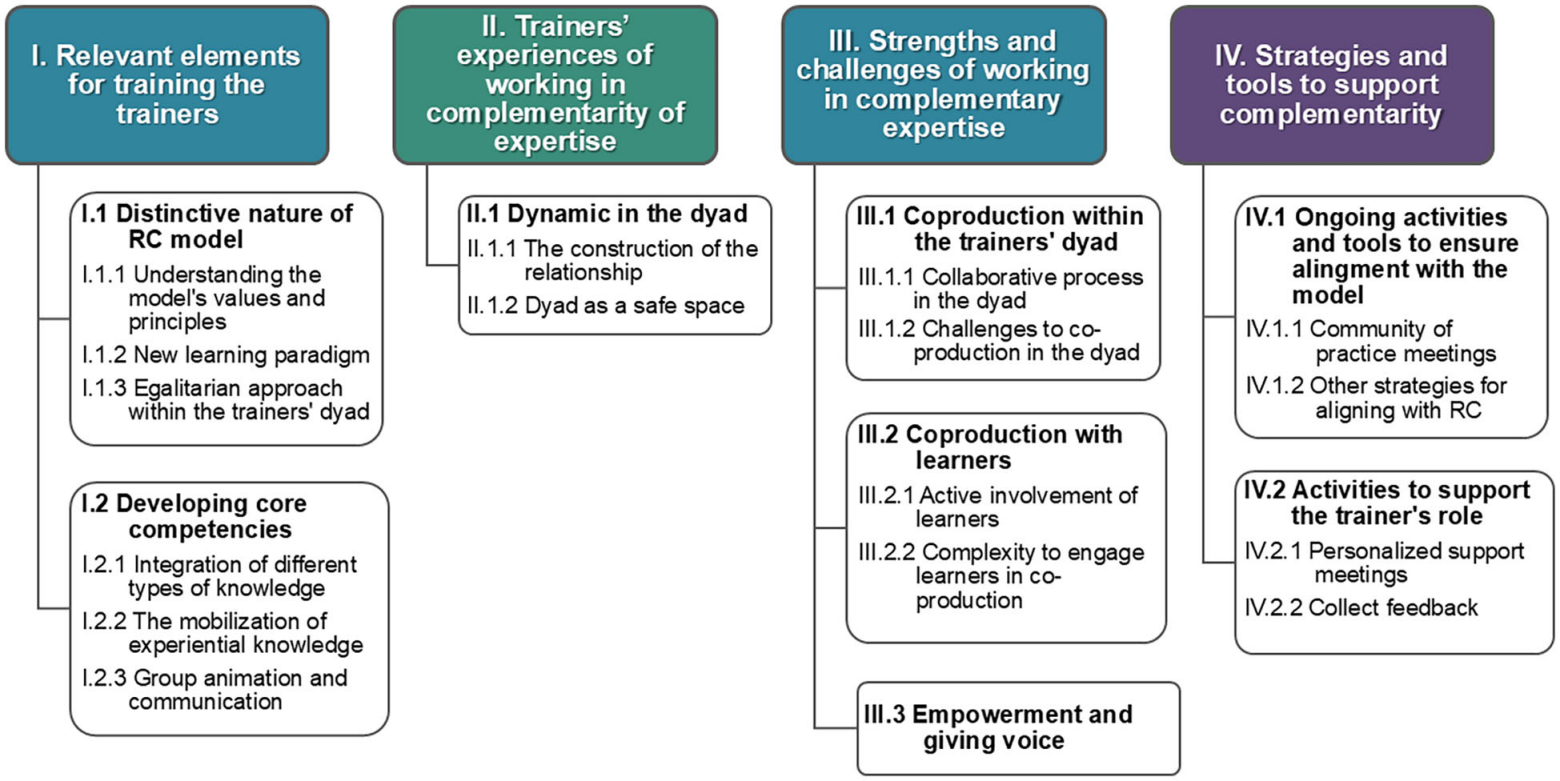
Themes emerged from the thematic analysis of the focus group transcripts.

This section presents the themes that emerged from the analysis of participants’ responses, as visually summarized in **Figure 1**. The themes are organized into sub-sections corresponding to the four research objectives. For each theme and corresponding sub-themes, relevant quotes from participants are included to illustrate and clarify the nuances of meaning. In presenting the results, an effort was made to select participants’ quotes, balancing Canadian and Italian contributions.

#### 3.2.1 First research objective: What are the relevant elements to be considered in the training of trainers?

Two themes (and six sub-themes) emerge from the analysis and enable us to identify the relevant elements to be considered in the training of trainers: the distinctive nature of the RC model (I.1) and the core competencies to be acquired and developed (I.2).These themes, and their respective sub-themes, emerged similarly in Quebec and in Lombardy.

##### 3.2.1.1 Distinctive nature of RC model (I.1)

Three sub-themes emerged from the participants’ narratives of the distinctive nature of the RC model. Participants emphasized the need for trainers to deeply understand the RC model, to be able to embody the model’s values and principles and apply them with conformity (I.1.1). They underlined that this process takes time.

The first thing to do is to understand what an RC is: acknowledge the reciprocity between different types of knowledge, define them clearly, and recognize that RCs are not support groups, but co-production groups. It takes time to really absorb and understand the model. [ … ] We could also talk about the values of RC and so on, [ … ] reflecting on the advantages of this model, what makes it different and innovative [ … ] trying to understand the added value of this model. P4 FG3.

Participants also mentioned the importance of introducing RC to trainers as a new learning paradigm that values the sharing and integration of diverse types of knowledge—experiential, theoretical, and clinical (I.1.2). Participants described this co-learning experience as “different” and “innovative”, with a particular influence on their personal or professional development.

One of the most important things to pass on is to explain that this is a completely different model, where all knowledge is valued [ … ] In the training of trainers, this must be emphasized, because we must completely change our way of learning, and therefore our way of facilitating the course. [ … ] It’s such a new learning paradigm. P1 FG1.

Another key aspect highlighted by participants is the egalitarian approach within the trainers’ dyad, described as essential for shaping the RC vision and promoting effective collaboration and co-production (I.1.3).

What seems most important to me is to emphasize the creation of an egalitarian relationship between all trainers, regardless of whether they bring theoretical or experiential knowledge. Everyone is considered equal. P2 FG3.

##### 3.2.1.2 Developing core competencies (I.2)

Three sub-themes emerged from the participants’ narratives describing the relevant competencies that trainers need to acquire and develop. The first is the competence of integrating different forms of knowledge: participants mentioned the importance of safeguarding the place of different types of knowledge (experiential, theoretical and clinical) and making connections between them, in order to create an integrated knowledge (I.2.1).

For me, integrating different types of knowledge is like tailoring, basting, embroidering, and holding multiple threads together. It is an interweaving of knowledge that occurs when different trainers with professional or experiential knowledge work together, and it is also the knowledge of the group of learners.P5 FG4.

The second sub-theme is the mobilization of experiential knowledge in a thoughtful and authentic way, ensuring that lived experience is valued (I.2.2). This competence consists of giving meaning and relevance to personal experiences and background, choosing and selecting which narratives can be shared during the RC course.

For me the important thing is sharing my experiential knowledge—what I have learned through my recovery experiences—and to make it as clear as possible to learners. P2 FG3.

The third sub-theme is group facilitation and communication (I.2.3). Group animation techniques and communication skills are considered valuable for increasing self-confidence in managing group dynamics and facilitating group discussion. Some participants mentioned the importance of adopting a facilitator attitude, which is better suited to stimulating exchanges and promoting knowledge integration among learners. Senior trainers are identified as key figures in shaping these competencies for new trainers.

It was very important for me to learn group techniques and know what activities to propose to engage people, as well as the language and communication to use with learners. [ … ] this has greatly improved my stress management during a course, and I have often learned some of these techniques from my partner with more expertise. P1 FG3.

#### 3.2.2 Second research objective: What is the trainers’ experience of working with complementary expertise?

One main theme (and two sub-themes) emerged from participants’ narratives allowing us to understand better trainers’ experiences of working with complementary expertise: the dynamic in the dyad (II.1). These themes, and their respective sub-themes, emerged in both samples.

##### 3.2.2.1 Dynamic in the dyad (II.1)

Two sub-themes emerged from participants’ narratives. The first, which emerged from participants’ accounts, is the construction of the relationship described as a progressive development of a meaningful personal relationship in the dyad (II.1.1).

It’s a relationship that must be built; it doesn’t just happen. Two people who have never worked together [ … ] you must be able to really succeed in creating this space for exchange. A relationship of mutual trust between people is needed. P3 FG2.

This relationship develops through mutual knowledge of each other’s backgrounds, personalities, interests, and values, within the context of a relational exchange that begins during the training of the trainers and continues thereafter.

I found these co-constructed meetings useful for getting to know people, but also for learning about their reality, their life stories or experiences, their interests [ … ]. P6 FG4.

The second sub-theme that emerged, equally important, is the dyad as a safe space (II.1.2). Participants emphasized the importance of experiencing a supportive relationship based on mutual trust, comfort, and non-judgmental support within the dyad. This environment allows trainers to express themselves freely, set their own tone, and feel emotionally supported.

So, a trainer needs to create this “bubble of trust” to feel comfortable and be in a safe space that allows us to talk about our vulnerabilities. [ … ] There are some people with whom chemistry and closeness arise spontaneously and the “bubble” works well [ … ] P4 FG1.

Furthermore, a secure relationship between trainers also helps to manage group dynamics and, in some cases, moments of tension with learners. Trainers often reported feeling a sense of mutual “protection” in these situations, emphasizing the importance of reciprocal support.

It is a co-construction that makes sharing possible, simultaneously exposing and protecting the person. P3 FG1

#### 3.2.3 Third research objective: What are the strengths and challenges of working with complementary expertise?

Three themes (and four sub-themes) emerged from the analysis, enabling us to identify the strengths and challenges of working with complementary expertise: co-production within the trainers’ dyad (III.1), co-production with learners (III.2), empowerment and giving voice (III.3).

##### 3.2.3.1 Co-production within the trainers’ dyad (III.1)

Two sub-themes emerge from the analysis of co-production within the trainers’ dyad. Firstly, in the trainer dyad, co-production is experienced as an ongoing collaborative process in which each trainer contributes their own knowledge—whether theoretical, clinical, or experiential—to shape the course content through continuous exchange and negotiations (III.1.1). This process is characterized by mutual recognition and respect for each other’s backgrounds, creating a shared space where differences are not only accepted but valued.

The most important thing for anyone who wants to contribute to an RC course is the desire to help and, with that, the willingness to take a step back, reconsider your point of view, and engage in genuine dialogue. It’s about being authentic. This is fundamental. P8 FG5.

Secondly, participants identified significant challenges to co-production within the dyad. Tensions or disagreements between trainers were seen as potential barriers to effective co-construction and co-facilitation, often arising from differences in personal style or divergent understandings of the RC model (III.1.2).

In dyads, the challenges I have faced often seem to be related to personalities, not just people’s knowledge. [ … ]. We are human beings, and sometimes something clicks with some people, and other times it doesn’t. During the co-construction of course ‘content, there is a lot of negotiation: we build and make decisions together [ … ]. With some people, this process works well, and with others it’s more difficult. And I think it’s not just a matter of type of knowledge, but also of chemistry between people. P1 FG1.

These difficulties were sometimes attributed to personality clashes or misaligned visions, reflecting deeper challenges in aligning perspectives and working collaboratively within the dyad.

I think the main difficulties are personality clashes and not sharing the same vision. Our first step will be to try to find that common vision. P3 FG1.

Some trainers (particularly in the Italian sample) provided some testimonials highlighting how difficulties in co-production seemed to be more frequent among new trainers with clinical or theoretical knowledge who already had pre-established content to include in the course.

Sometimes it has been challenging when there is no complete agreement on objectives and methods, or when you realize that you come from very different backgrounds and sometimes you must be patient and accept that not everyone has followed the same path. However, you also learn to adapt and appreciate people and the context. P6 FG4.

Furthermore, when trainers with clinical or theoretical backgrounds adopted a rigid stance not always coherent with RC, this was perceived as limiting the integration, which overshadowed the contributions of trainers with experiential knowledge.

I encountered some difficulties when there was no equality of roles in the dyad. There was an imbalance that meant that one trainer took up more space during the course. I perceived this as a kind of hierarchy. He didn’t do it to steal the show, but he did it anyway. P8 FG5.

##### 3.2.3.2 Co-production with learners (III.2)

Beyond the dyad, participants emphasized the importance of involving learners as active contributors to the co-production process. Two sub-themes emerge from the analysis of this theme. Firstly, participants reported that co-production does not end with the dyad but continues as an ongoing and dynamic process that includes learners (III.2.1).

I also realized that letting students talk and giving them space is interesting. They don’t feel like they’re sitting behind a screen watching but want to keep sharing ideas with each other. This [ … ] makes the interaction really interesting. P4 FG1.

The outcome of the co-production process is never the same in every course, but can vary depending on the group, as learners bring different levels of involvement and personal contribution.

The course we offer will never be exactly the same, because each group of learners is different [ … ]. Each time, we co-construct it with new learners, and the result is slightly different from the previous one. P2 FG1.

Some trainers (particularly in the Italian sample) emphasized the importance of managing uncertainty and adapting to specific group dynamics, since the outcome of co-production is not considered something that can be planned.

However, integrating [different types of knowledge] means that you don’t know in advance what the result will be, precisely because the result is the fruit of this indeterminacy and complementarity. So, this is the aspect that I think is important to emphasize: accepting this indeterminacy. P2 FG4.

Secondly, according to participants, involving learners in co-production can also present some complexities (III.2.2). Trainers reported some challenges, such as stimulating active participation in group discussions on mental health issues or helping learners understand the RC model, especially when it does not align with their expectations.

During the last course, a family member expressed strong anger, and we felt a bit like there was an elephant in the room. There was a risk of focusing solely on that feeling. [ … ] Some of the most challenging aspects for learners often relate to managing their expectations. Addressing these challenges sometimes requires teamwork among trainers to help redefine or manage these expectations in a constructive way. P5 FG4.

In addition, some participants reported difficulties in managing emotions and certain demanding attitudes of some learners which were not aligned with co-production stance.

The first session can actually be unsettling, especially when you are not used to attending a training course where people actively talk and express their knowledge. P1 FG3.

##### 3.2.3.3 Empowerment and giving voice (III.3)

Despite these challenges, participants with lived experience described their involvement in RC as deeply rewarding. Becoming trainers contributed to their recovery, improved their self-esteem, and helped them overcome social isolation, enabling them to find their “place” in the community.

For those who have experienced mental health difficulties and are on the road to recovery, the impact is enormous. Expressing oneself is fundamental. Regaining self-determination, empowerment, rebuilding self-confidence, overcoming self-stigmatization [ … ]. And then there’s boosting self-esteem, breaking out of isolation, getting active again, participating in social life, feeling like you’re contributing: for me, it’s been extremely meaningful. P2 FG3.

They emphasized the importance of giving voice to their experiences, not only for their own personal validation, but also as a means of promoting cultural and institutional change. Trainers, including those with theoretical and clinical knowledge, believed that their role could contribute to reducing stigma by seeking to influence the practice of mental health professionals and promoting a more supportive and non-judgmental attitude towards mental illness.

Working in RC as a trainer meant questioning the meaning of our rehabilitation activities [ … ] in terms of the possibility of transforming communities, the way communities see and judge people with mental health problems, as this is one of the most complex elements to address and transform. [ … ] It was therefore an opportunity to reflect on what my services do. P4 FG4.

#### 3.2.4 Fourth research objective: What are the strategies and tools to support complementarity of expertise?

Two main themes (and four subthemes) emerged concerning strategies and tools to support complementarity of expertise: ongoing activities and tools to ensure alignment with the model (IV.1) and activities to support the trainer’s role (IV.2).

##### 3.2.4.1 Ongoing activities and tools to ensure alignment with the model (IV.1)

Participants identified several activities and support strategies that contribute to strengthening alignment with the RC model and supporting trainers in their role. Two sub-themes emerge from the analysis. Firstly, the community of practice meetings were widely appreciated as opportunities for continuous learning, peer exchange, and mutual support (IV.1.1). These meetings foster a climate in which trainers can reflect on their role, explore strategies for addressing challenges, and gain new perspectives, while reducing feelings of isolation. These meetings also provide an opportunity to revisit the core principles and values of RC and maintain fidelity to the model.

Being part of a community of practice means actively participating. These are spaces where I can reflect, talk about my experiences, exchange ideas, and listen to other people’s points of view. [ … ] we’re together, we exchange ideas, we learn from each other. If we don’t take advantage of these opportunities to train as trainers, I think we’re missing out on something. P1 FG1.

Secondly, some participants (particularly in the Quebec sample) reported other strategies for aligning trainers with the RC (IV.1.2). The pre-selection interview was identified as a key step in orienting new trainers to the values of the RC and exploring expectations Also, co-facilitation with a senior trainer (or a different trainer from the previous dyad) was highlighted as an important learning opportunity to experience how values and principles are embodied in the role of the trainer.

One strategy for staying aligned with the model is to change the dyad. Over time, I have worked with different people, and each time I have had to remind myself of the principles and values of the model. I think this is a good way to stay grounded. P4 FG1.

In addition, in Quebec, participants mentioned self-appraisal tools that aim to promote understanding of key principles and mechanisms of action of the RC model, stimulating self-reflection on one’s own competencies and role as a trainer.

At CASR, we have developed a self-appraisal tool [ … ] that provides food for thought for each trainer. Personally, I feel that I still need to understand how to use it properly and take a further step forward in my journey as an RC trainer. P1 FG2.

##### 3.2.4.2 Activities to support the trainer’s role (IV.2)

Two sub-themes emerge from the analysis. Firstly, activities to support the trainer’s role were considered essential both during the co-construction of the course and to facilitate the delivery of the RC course (IV.2.1). The personalized support meetings provided by CASR staff were appreciated for offering both practical guidance and relational support, especially in addressing interpersonal challenges within the dyad. These meetings helped to manage trainer expectations and ensure comfort within the trainer dyad, with the aim of creating fertile ground for improving relationships and making them enjoyable, safe, and trust-based.

There are so many factors that can influence how things turn out. I think it’s so important to maintain open communication with the support team. That’s why we’re here for: we’re committed to making everything work and ensuring that our trainers feel as comfortable as possible. That’s why we offer technical support [ … ] and we aim to eliminate as many stress factors as possible. P1 FG3

Secondly, participants emphasized the importance of gathering their feedback, assessing their satisfaction, identifying strengths and challenges, as well as areas for future improvement (IV.2.2.). Strategies such as trainer satisfaction questionnaires or periodic feedback sessions were suggested as useful resources.

We always collect satisfaction questionnaires from learners, but it might also be interesting to collect them from trainers as well [ … ] A survey on trainers’ experience and satisfaction regarding strengths and difficulties encountered could be an idea to develop. P6 FG4.

## 4 Discussion

The objectives of the present study were to: (1) identify the most relevant elements to be considered in the training of trainers; (2) explore trainers’ experiences of working with complementary expertise; (3) examine the strengths and challenges; (4) identify strategies and tools that can support complementarity of expertise. Regarding the first objective, participants identified the distinctive nature of the RC model that should be considered in the training of trainers. These included both core principles, such as co-production and egalitarianism, and relevant competencies, particularly the ability to integrate different forms of knowledge, mobilize lived experiences, and facilitate inclusive group dynamics and communication. These two levels, principles and competencies, are closely related, as competencies represent the concrete application of principles. This new learning paradigm, based on the integration of different types of knowledge, differentiates the RC model from other approaches (1). Furthermore, participants not only emphasized the importance of effectively conveying both fundamental principles and related competencies, but also reported concrete strategies for promoting them, helping to bridge the gap reported by Dalgarno and colleagues (11), who called for concrete examples of how such competencies can be developed in practice. These findings also complement a recent contribution (21), which proposed a detailed training program implemented in 2019 on key competencies in various operational areas, including training activities on mobilization and integration of diverse knowledge.

Moving on to the second objective, participants emphasized the importance of cultivating a meaningful relationship within the dyad over time and promoting a safe environment that allows for emotional communication and supports the co-construction of knowledge and practices. The way participants describe the relationship within the trainer dyad is consistent with previous research that has examined and conceptualized the supportive relational climate that fosters co-production processes (20, 26–28). The relational climate in the dyad is characterized by mutual support, non-judgment, and an egalitarian approach that goes beyond clinical, theoretical, or experiential knowledge. It can therefore be said that the relationship between trainers serves as a relational model in the courses as well, both between trainers and learners and among learners themselves. In this sense, the dyadic relationship not only promotes co-facilitation but also embodies the fundamental principles and values of the RC model, as previously stated by Toney and colleagues (6).

Regarding the third objective, participants reported on the strengths and challenges of working with complementary types of knowledge. Co-production was described as a dynamic and collaborative process in which everyone engages in constructive dialogue and makes contributions that enrich the learning process. Participants defined co-production as a process that begins within the dyad and continues actively with the learners. This description of co-production reflects definitions found in the literature, which frame it as a process based on equal, respectful, and constructive relationships (3, 9). Although participants emphasized the importance of co-production as a process based on equality and mutual respect, they also acknowledged that significant challenges need to be addressed. Regarding trainers, difficulties often arise related to personality compatibility and varying degrees of willingness to collaborate and authentically embrace the values and principles of RC. In some cases, these challenges may be linked to an incomplete or partial understanding of the RC model itself, a factor already noted in recent literature (11, 19). Furthermore, another challenge reported by participants concerns the relationship between trainers and learners: learners may express disagreement and tension or request a level of emotional support that is not in line with the context of RC. Mismatched expectations can partly explain this, some learners may approach RC courses with assumptions closer to therapeutic contexts. The role of the trainer, therefore, is both to recognize and contain these emotional responses and to clearly redefine the purpose of RC: as space for active learning, personal growth and empowerment (29–31). The challenges are closely linked to what was discussed earlier regarding the dyadic experience: if a constructive relational dynamic is not established between trainers, it is difficult to achieve collaborative co-production. It is therefore essential to support trainers both in managing their relationships and in dealing with the tensions and emotional demands of learners.

Finally, in response to our last objective, participants proposed strategies and tools to support complementarity. Two main groups of resources were identified: the first includes some strategies to align with the RC model such as communities of practice meetings, self-reflection tools, and the dyad change. The second category includes some activities proposed by the RC staff to support trainer’s role, such as personalized support in response to the complex dynamics that can arise between trainers and learners. These resources were considered valuable for maintaining alignment with RC principles, encouraging reflective practice, and promoting the well-being of trainers. These findings align with existing literature on co-production, which emphasizes the importance of providing ongoing guidance and support to trainers to ensure a shared understanding and commitment to the values of co-production (11). Our results add further possible strategies to those already mentioned in the literature, such as training activities in small groups (32, 33), mutual support during informal debriefings (34, 35) and in group or individual supervision (11).

Although the research was not intended to compare the two Recovery Colleges, some distinctive features emerged. In the Italian sample, co-production within the dyad and with learners is described as a process that involves the ability to tolerate uncertainty. The importance of managing uncertainty and adapting to the unpredictable contributions and dynamics of the group is a strategy to be adopted because the outcome of co-production is not considered something that can be planned. In contrast, the Quebec sample showed greater attention to developing competencies in co-constructing self-observation and self-reflection tools, as well as designing and implementing a detailed training program for new trainers. In addition, participants emphasized the usefulness of complementary strategies, such as pre-selection interviews and the reconfiguration of dyads to ensure better alignment with the RC model and the expectations of new trainers.

### 4.1 Limitations and future perspectives

The primary limitation of this study pertains to the sampling and generalizability of the results. Although the research team sought to include trainers with different profiles working in two different sites, the results obtained refer only to these two specific contexts.

Further research could focus on evaluating the impact of training and support programs for RC trainers on fidelity to RC model as well as the development of recommendations for the design and implementation of training programs to support the complementarity of expertise. Moreover, our study also suggests the importance of conducting possible future multicenter and cross-country studies to explore the influence of the local context on the implementation of the RC model. This also aligns with the conclusion of a recent systematic review of RC studies (22), which highlights the need for more studies and international collaborations between different RCs.

## 5 Conclusion

To our knowledge, this is the first article that focuses entirely on the experience of RC trainers to explore the topic of knowledge complementarity, using a qualitative methodology and a multicenter approach. The results suggest the importance of raising awareness among trainers about the distinctive nature of the RC model and paying attention to how it is applied in practice.

Starting from the relationship between trainers in the dyad, it is therefore important to foster egalitarian and supportive relationships, as these can serve as a model for co-production during RC courses. Finally, to improve the co-production process, trainers need access to regular community of practice meetings and other opportunities for continuous learning. Based on these suggestions, which stem from the direct experience of trainers, future research will therefore aim to extend these findings to develop guidelines and training materials.

## Data Availability

The raw data supporting the conclusions of this article will be made available by the authors, without undue reservation.

## References

[1] Perkins R Repper J . When is a recovery college not a recovery college? Ment Health Soc Incl. (2017) 21:65–72. doi: 10.1108/MHSI-02-2017-0005

[2] Hayes D Camacho EM Ronaldson A Stepanian K McPhilbin M Elliott RA . Evidence-based Recovery Colleges: developing a typology based on organisational characteristics, fidelity and funding. Soc Psychiatry Psychiatr Epidemiol. (2023) 59:1–10. doi: 10.1007/s00127-023-02452-w, PMID: 36905435 PMC10007645

[3] King T Meddings S . Survey identifying commonality across international Recovery Colleges. Ment Health Soc Incl. (2019) 23:121–8. doi: 10.1108/MHSI-02-2019-0008

[4] Crowther A Taylor A Toney R Meddings S Whale T Jennings H . The impact of Recovery Colleges on mental health staff, services and society. Epidemiol Psychiatr Sci. (2019) 28:481–8. doi: 10.1017/S204579601800063X, PMID: 30348246 PMC6998922

[5] Sommer J Gill K Stein-Parbury J . Walking side-by-side: Recovery Colleges revolutionising mental health care. Ment Health Soc Incl. (2018) 22:18–26. doi: 10.1108/MHSI-11-2017-0050

[6] Toney R Knight J Hamill K Taylor A Henderson C Crowther A . Development and evaluation of a recovery college fidelity measure. Can J Psychiatry. (2019) 64:405–14. doi: 10.1177/0706743718815893, PMID: 30595039 PMC6591755

[7] Anfossi A . The current state of recovery colleges in the UK. In: Nottingham: implementing recovery through organisational change. Nottingham, UK: ImRoc (2017).

[8] Dalgarno M Oates J . The meaning of co-production for clinicians: an exploratory case study of practitioner trainers in one Recovery College. J Psychiatr Ment Health Nurs. (2018) 25:349–57. doi: 10.1111/jpm.12469, PMID: 29763995

[9] Rethink Group. In: Co-production in commissioning: getting started. Available online at: https://www.rethink.org/media/2256/co_production_getting_started_guide.pdf (Accessed November 21, 2025).

[10] Perkins R Repper J Rinaldi M Brown H . Recovery colleges. London: Centre for Mental Health (2012).

[11] Dalgarno M Foye U Oates J Leamy M . How has co-production been used to design and deliver Recovery College courses? A scoping review of guidance, training and experience of trainers. Health Educ J. (2025) 84:542–557. doi: 00178969251327658

[12] Morgan S Rose S Dyer R . Co-Production. The Essential Component in Recovery Colleges. Cheltenham: Severn and Wye Recovery College (2018).

[13] Pledger M . The value of lived experience: Co-production and collaboration in Recovery Colleges. Journal of Recovery in Mental Health. (2018) 1:21–28.

[14] Whish R . Peer support and debriefing practices in Recovery Colleges. Psychiatr Rehabil J. (2021) 44:257–65. doi: 10.1108/JMHTEP-11-2021-0130

[15] Gill KH . Recovery Colleges. Co-production in action: The value of the lived experience in ‘Learning and Growth for Mental Health’. Health Issues. (2014) 113:10–14.

[16] Meddings S Byrne D Barnicoat S . Co-delivered and co-produced: Creating a recovery college in partnership. The Journal of Mental Health Training, Education and Practice (2024) 9:16–25. doi: 10.1108/JMHTEP-04-2013-0011

[17] Lewis A King T Herbert L Repper J . Co-production—sharing our experiences, reflecting on our learning. In: ImROC—Implementing recovery through organisational change. Nottingham, UK: ImRoc (2017).

[18] West J Birt L Wilson D . A case study of co-production within a mental health Recovery College dementia course: Perspectives of a person with dementia, their family supporter and mental health staff. Frontiers in Rehabilitation Sciences (2022) 3:920496. doi: 10.3389/fresc.2022.920496, PMID: 36188994 PMC9397742

[19] Bester KL McGlade A Darragh E . Is co-production working well in recovery colleges? Emergent themes from a systematic narrative review. J Ment Health Train Educ Pract. (2021) 17:48–60. doi: 10.1108/JMHTEP-05-2021-0046

[20] Doroud N King A Zirnsak TM Brasier C Hall T Jordan H . Creating an oasis of hope, inclusion and connection: students and stakeholders’ experiences of a pilot Recovery College. J Ment Health. (2024) 33:92–100. doi: 10.1080/09638237.2023.2245881, PMID: 37641410

[21] Vallarino M Briand C Lord MM Sauvageau A . Logic model for a train-the-trainer program ensuring alignment with recovery college principles and values. Ment Health Soc Incl. (2025). doi: 10.1108/MHSI-02-2025-0057

[22] Briand C Vallée C Luconi F Thériault J Sauvageau A Bellemare J . State-of-the-art literature review of Recovery College evaluative studies between 2013–2024. Front Psychiatry. (2025) 16:1584110. doi: 10.3389/fpsyt.2025.1584110, PMID: 40831527 PMC12358375

[23] Zabel E Donegan G Lawrence K French P . Exploring the impact of the Recovery Academy: a qualitative study of Recovery College experiences. J Ment Health Train Educ Pract. (2016) 11:162–71. doi: 10.1108/JMHTEP-12-2015-0052

[24] Martin K Stevens A Arbour S . The process of developing a co-design and co-delivery initiative for mental health programming. J Psychosoc Rehabil Ment Health. (2017) 4:247–51. doi: 10.1007/s40737-017-0091-z

[25] Miles MB Huberman AM . Qualitative data analysis: An expanded sourcebook. 2nd ed. Thousand Oaks, CA: Sage (1994).

[26] Briand C Hakin R Macario de Medeiros J Luconi F Vachon B Drolet MJ . Learner experience of an online co-learning model to support mental health during the COVID-19 pandemic: a qualitative study. Int J Environ Res Public Health. (2023) 20:2498. doi: 10.3390/ijerph20032498, PMID: 36767864 PMC9915127

[27] Toney R Elton D Munday E Hamill K Crowther A Meddings S . Mechanisms of action and outcomes for students in Recovery Colleges. Psychiatr Serv. (2018) 69:1222–9. doi: 10.1176/appi.ps.201800283, PMID: 30220242

[28] Thompson H Simonds L Barr S Meddings S . Recovery colleges: long-term impact and mechanisms of change. Ment Health Soc Incl. (2021) 25:232–242. doi: 10.1108/MHSI-01-2021-0002

[29] Muir-Cochrane E Lawn S Coveney J Zabeen S Kortman B Oster C . Recovery college as a transition space in the journey towards recovery: an Australian qualitative study. Nurs Health Sci. (2019) 21:523–30. doi: 10.1111/nhs.12637, PMID: 31495060

[30] Omundo J Stiehl SA Schulz M Zingsheim A Schmedthenke S Löhr M . Examining Recovery College experiences: perspectives on learning and personal growth from an online survey. Psychosoc Rehabil Ment Health. (2025), 1–11. doi: 10.1037/h0095655

[31] Selbekk AS Kvelland LT Nordås R Kvia A Robertson IE . A place without walls, only opportunities: personal accounts of attending Recovery Colleges in Norway. Front Psychiatry. (2023) 14:1233598. doi: 10.3389/fpsyt.2023.1233598, PMID: 37965357 PMC10642191

[32] Ali I Benkwitz A McDonald P . Setting up a Recovery College: exploring the experiences of mental health service-users, staff, carers and volunteers. J Psychosoc Rehabil Ment Health. (2023) 10:157–66. doi: 10.1007/s40737-022-00295-3

[33] Morgan AJ Reavley NJ Ross A Too LS Jorm AF . Interventions to reduce stigma towards people with severe mental illness: systematic review and meta-analysis. J Psychiatr Res. (2018) 103:120–33. doi: 10.1016/j.jpsychres.2018.05.017, PMID: 29843003

[34] Dalgarno M Oates JL . The meaning of co-production for clinicians: an exploratory case study of Practitioner Trainers in one Recovery College. J Psychiatr Ment Health Nurs. (2018) 25:349–57. doi: 10.1111/jpm.12469, PMID: 29763995

[35] Whish R Huckle C Mason O . What is the impact of recovery colleges on students? A thematic synthesis of qualitative evidence. J Ment Health Train Educ Pract. (2022) 17:443–54. doi: 10.1108/JMHTEP-11-2021-0130

